# Identification and application of piwi-interacting RNAs from seminal plasma exosomes in *Cynoglossus semilaevis*

**DOI:** 10.1186/s12864-020-6660-7

**Published:** 2020-04-15

**Authors:** Bo Zhang, Na Zhao, Lei Jia, Jinyuan Che, Xiaoxu He, Kefeng Liu, Baolong Bao

**Affiliations:** 1 Key Laboratory of Exploration and Utilization of Aquatic Genetic Resources (Shanghai Ocean University), Ministry of Education; International Research Center for Marine Biosciences at Shanghai Ocean University, Ministry of Science and Technology; National Demonstration Center for Experimental Fisheries Science Education, Shanghai Ocean University, Shanghai, 201306 China; 2 Tianjin Sea Fisheries Research Institute, Tianjin, China; 3 Tianjin Medicine Biotechnology Co, Ltd, Tianjin, China

**Keywords:** Piwi-interacting RNAs, Seminal plasma exosome, *Cynoglossus semilaevis*, Pseudomale, Biomarkers

## Abstract

**Background:**

Piwi-interacting RNAs (piRNAs) have been linked to epigenetic and post-transcriptional gene silencing of retrotransposons in germ line cells, particularly in spermatogenesis. Exosomes are important mediators of vesicle transport, and the piRNAs in exosomes might play an important role in cell communication and signal pathway regulation. Moreover, exosomic piRNAs are promising biomarkers for disease diagnosis and physiological status indication. We used *Cynoglossus semilaevis* because of its commercial value and its sexual dimorphism, particularly the sex reversed “pseudomales” who have a female karyotype, produce sperm, and copulate with normal females to produce viable offspring.

**Results:**

To determine whether piRNAs from fish germ line cells have similar features, seminal plasma exosomes from half-smooth tongue sole, *C. semilaevis*, were identified, and their small RNAs were sequenced and analysed. We identified six signature piRNAs as biomarkers in exosomes of seminal plasma from males and pseudomale *C. semilaevis*. Bioinformatic analysis showed that all six signatures were sex-related, and four were DNA methylation-related and transposition-related piRNAs. Their expression profiles were verified using real-time quantitative PCR. The expression of the signature piRNAs was markedly higher in males than in pseudomales. The signature piRNAs could be exploited as male-specific biomarkers in this fish.

**Conclusions:**

These signatures provide an effective tool to explore the regulatory mechanism of sex development in *C. semilaevis* and may provide guidance for future research on the function of piRNAs in the generative mechanism of sex reversed “pseudomales” in *C. semilaevis*.

Half smooth tongue sole (*Cynoglossus semilaevis*), a commercially valuable flatfish that is widely distributed in Chinese coastal waters, is commonly found in shallow waters on a muddy or sandy bottom [1]. Many previous studies have shown that *C. semilaevis* employs a heterogametic sex determination system (ZW/ZZ) [2, 3] and has significant sexual dimorphism, with a larger female body size and faster growth rate [4, 5]. This species is attracting more attention in reproductive and sex-related research, and could become a tool to study sex determination in fish [6]. Furthermore, this species also exists as pseudomale fish, both in nature and aquaculture [7]. The high proportion of males in populations of *C. semilaevis* was partly attributed to the considerable number of pseudomales. The pseudomale has the same karyotype as the female fish but has the physiological characteristics of males [8]. Interestingly, pseudomale fish are fertile and can pass on their pseudomale characteristic to their offspring. When pseudomale fish are used as parents, an imbalance in the proportion between the female and male tongue soles will arise [9]. Distinguishing pseudomale from male fish and inhibiting them from mating with females could maintain the sex balance in *C. semilaevis* populations, which has great commercial value in aquaculture [10]. In addition, it is important to explore the influencing factors and determining mechanism of pseudomale occurrence to obtain further details on the sex determination mechanism of fish.

Piwi interacting RNAs (piRNAs) are single-stranded, 25- to 33 -nt-long small RNAs that function via forming RNA-protein complexes through interactions with piwi proteins [11]. PiRNAs are distinct from microRNAs (miRNAs) in terms of their size (26–31 nt rather than 21–24 nt), lack of sequence conservation, and increased complexity [12]. Previous profiling studies showed that miRNAs are widely expressed in different tissues, while piRNAs are abundant in gametes [13, 14]. PiRNAs have been found in the testes and ovaries in mammals [15], and were detected in both male and female germlines [16, 17]. PiRNAs play roles in spermatogenesis in *Caenorhabditis elegans*, mice, and humans. The piRNA pathway relies on the specificity provided by the piRNAs to identify transposon element (TE) targets, while the effector function is provided by the piwi protein. Different piRNAs recognize different TE target gene sequences to play different regulatory roles [18]. We hypothesized that piRNAs might be differentially expressed in germlines between males and pseudomales in *C semilaevis*, and might play a role in the cross generation inheritance of pseudomales. If so, how are piRNAs, as cross-generational sex-related regulatory factors, stably transmitted? Consequently, we investigated piRNAs in exosomes from the seminal fluid on *C. semilaevis*. Previously, small RNAs, including piRNAs in exosomes from body fluid, have been reported as biomarkers [19, 20]. Exosomes from mouse and human seminal fluid have been isolated and analysed, representing an excellent system to study piwi-interacting genes and their regulatory network [21]. Although several different kinds of sex molecular markers have been developed in *C. semilaevis*, such as amplified fragment-length polymorphism (AFLP) markers [3], co-dominant microsatellite markers [22], single nucleotide polymorphisms (SNPs) [23], and miRNAs markers [24], identifying different types of sex markers is also important to study the sex determination mechanism of this fish. In the present study, we compared the piRNA profiles in exosomes from male and pseudomale seminal plasma to identify the differentially expressed piRNAs. PiRNAs with significant differential expression between males and pseudomales were selected from candidate piRNAs for sex identification and their expression profiles were verified using quantitative real-time reverse transcription PCR (qRT-PCR). The signatures could be used as biomarkers to distinguish pseudomale from male fish in *C. semilaevis*. In addition, the interaction and regulatory mechanism between piRNAs and target genes would play an important role in explaining the cross generational genetic mechanism of pseudomale *C semilaevis*.

## Results

### Identification and characterization of exosomes

**Fig. 1:**
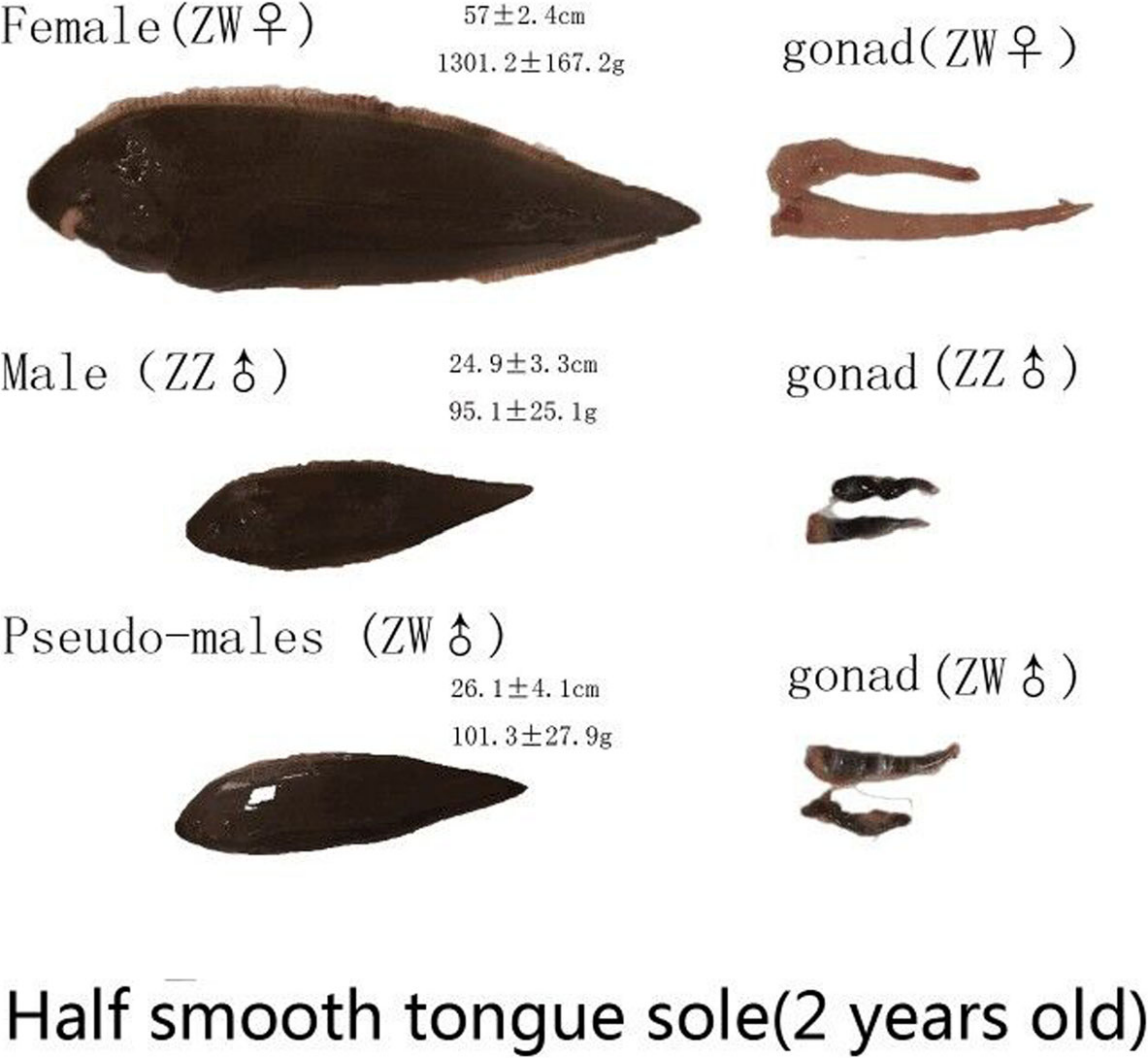
Morphology of half-smooth tongue sole and its gonads. Images of a female, a normal male, and a pseudomale at 2 years of age

**Fig. 2:**
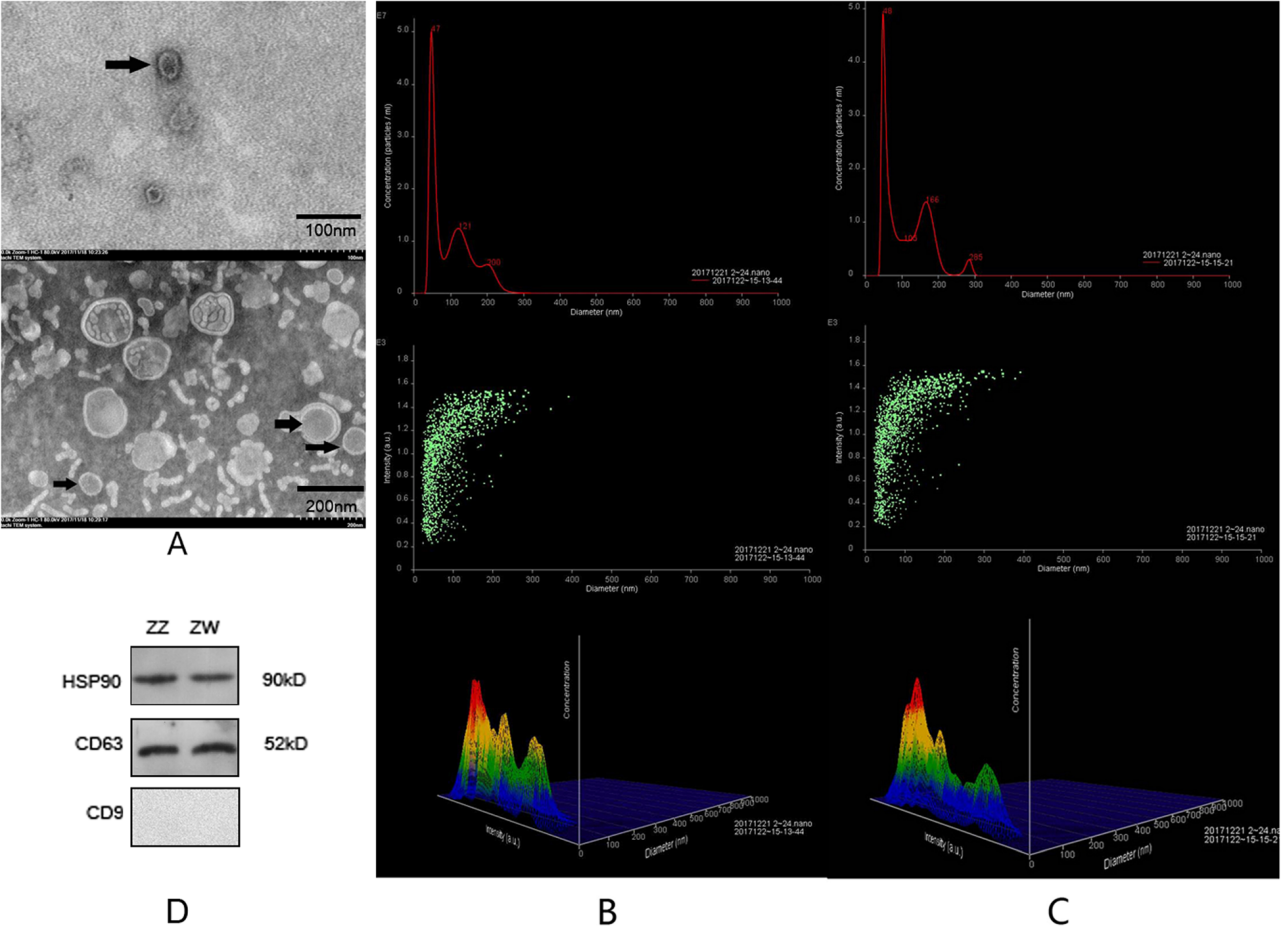
Isolation and identification of exosomes from *C. semilaevis* seminal plasma. **a** Electron microscope images of exosomes; (**b**) Particle size distributions and concentration of exosomes in males analysed using NTA 2.3; Top: line chart; middle: scatter diagram; bottom: three-dimensional graph; (**c**) Particle size distributions and concentration of exosomes in pseudomales analysed using NTA 2.3; Top: line chart; middle: scatter diagram; bottom: three-dimensional graph; (**d**) Western blotting for CD63, heat shock protein 90 (HSP90), and CD9

We collected about 20 ml of seminal plasma from 60 male and 60 pseudomale donors, respectively (Fig. 1). Exosomes were isolated and purified using an SBI ExoQuick-TC kit after the samples were filtered through a 0.45-μm membrane. We used transmission electron microscopy (TEM) and nanoparticle tracking analysis (NTA) to identify exosomes and capture images and data for granularometric analysis of the exosome preparations (Fig. 2a). The exosome particles had diameters ranging from 30 to 150 nm, which was consistent with the characteristic size range (30–120 nm) of exosomes, and represented almost 70% (67.043 and 69.693%) of all the particles in both samples, with mean sizes of 108.2 ± 0.57 nm and 116.3 ± 0.32 nm in males and pseudomales, respectively. The main peaks of particle size in the NTA analysis were 47 and 48 nm, respectively. The concentrations of two samples were 24.9 ± 0.94 × 10^8^ and 23.9 ± 2.64 × 10^8^ particles/ml, respectively. (Fig. 2b and c). All these measurements suggested that the exosome preparations isolated from male and pseudomale *C. semilaevis* seminal plasma contain a heterogeneous mixture of exosomes and microvesicles, which was similar to that in previous reports [24].

We also investigated the presence of three tetraspanins as exosome markers using western blotting, including CD63, CD9, and heat shock protein 90 (HSP90) to confirm the existence of exosomes. Immunoreactive bands corresponding to CD63 and HSP90 were observed, whereas CD9 had no obvious immunoreactive band (Fig. 2d and Supplementary Figs. 1–3). These results were in line with those of previous studies of exosomes from the serum of *C. semilaevis*^24^.

#### Small RNA sequencing and the nucleotide composition in the exosomes

**Fig. 3:**
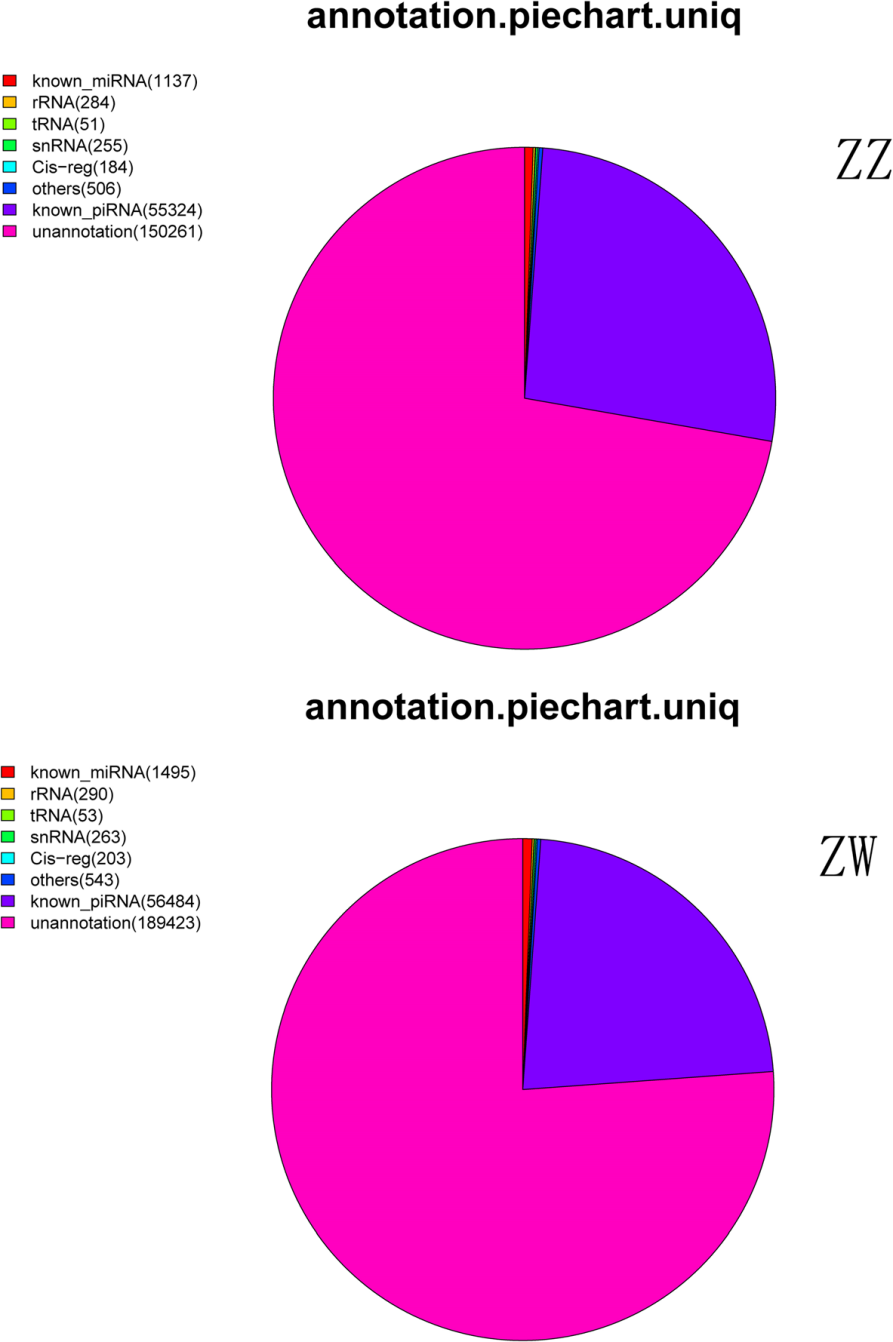
Pie charts of the classification and annotation of the unique reads of each donor group. Top: samples from pseudomale (ZW♂) *C. semilaevis* donors; bottom: samples from male (ZZ♂) *C. semilaevis* donors. MiRNA, microRNA; rRNA, ribosomal RNA; tRNA; transfer RNA; snRNA, small nuclear RNA; piRNA, piwi interacting RNA

RNA was isolated from the exosome preparations from male (ZZ♂) and pseudomale (ZW♂) *C. semilaevis* and sequenced for small RNA analysis. The reads that aligned to the genome of half-smooth tongue sole were employed to determine the length distribution of the two groups: we found that the peak values of both groups were mainly concentrated at 31 bp, which corresponded with the characteristic length of piRNAs (Supplementary Fig. 4). The numbers of different types of mature piRNAs for the species were calculated as follows: the number of unique known piRNA aligned reads was 56,484, representing about 22.71% of all clean reads in the pseudomale donor group, while 55,324 (26.6%) came from male donors. We constructed pie charts for the classification and annotation of the small RNA reads of each donor group (Fig. 3)... The novel piRNAs was predicted using RNAplex. We employed the unaligned sequences filtered from piRBase to carry out novel piRNA prediction. The predicted novel piRNAs were between 21 and 38 bp and could be mapped to the genome. In total, 14,006 non-repetitive novel piRNAs were predicted from both pseudomales (ZW♂) and male (ZZ♂) donors (Additional file 1). We also obtained the number of novel piRNA categories in both donor groups: 7070 in ZZ♂ (Additional file 2) donor group and 11,588 in ZW♂ (Additional file 3) donor group eliminating 4652 repetitive piRNAs.

### Identification of signature piRNAs between male and pseudomale *C. semilaevis*

The differential expression profiles of piRNA were investigated between male and pseudomale *C. semilaevis* using the TPM (transcript per million) values. In total, 26,135 differentially expressed piRNAs were identified according to the criteria detailed in the methods section (Additional file 4). Among these piRNAs, 15,373 were upregulated and 10,762 were downregulated in ZZ♂ compared with ZW♂. Using further screening conditions, we filtered out the novel piRNAs (6622) because of their unproven existence and narrowed the highly expressed piRNAs down to 87 known piRNAs under the condition of at least a TPM of one group ≥150 and a fold-change (ZZ♂ / ZW♂) ≥ 100. Then, considering that 87 was too many piRNAs for subsequent analysis, we adjusted the fold-change (ZZ♂ / ZW♂) to ≥200 and at least a TPM of one group ≥400, which narrowed the dataset 44 candidate piRNAs (Supplementary Fig. 5). We predicted the target genes of the 44 candidate signature piRNAs that were differentially expressed in males and pseudomales. In the present study, “signature” meant a piRNA marker with significant differential expression as verified by qRT PCR. After piRNA-target prediction, we obtained 12,145 piRNA-target pairs and 6231 target genes (Additional file 5).

#### Target prediction for candidate signature piRNAs and GO enrichment and KEGG pathway enrichment analysis

**Fig. 4:**
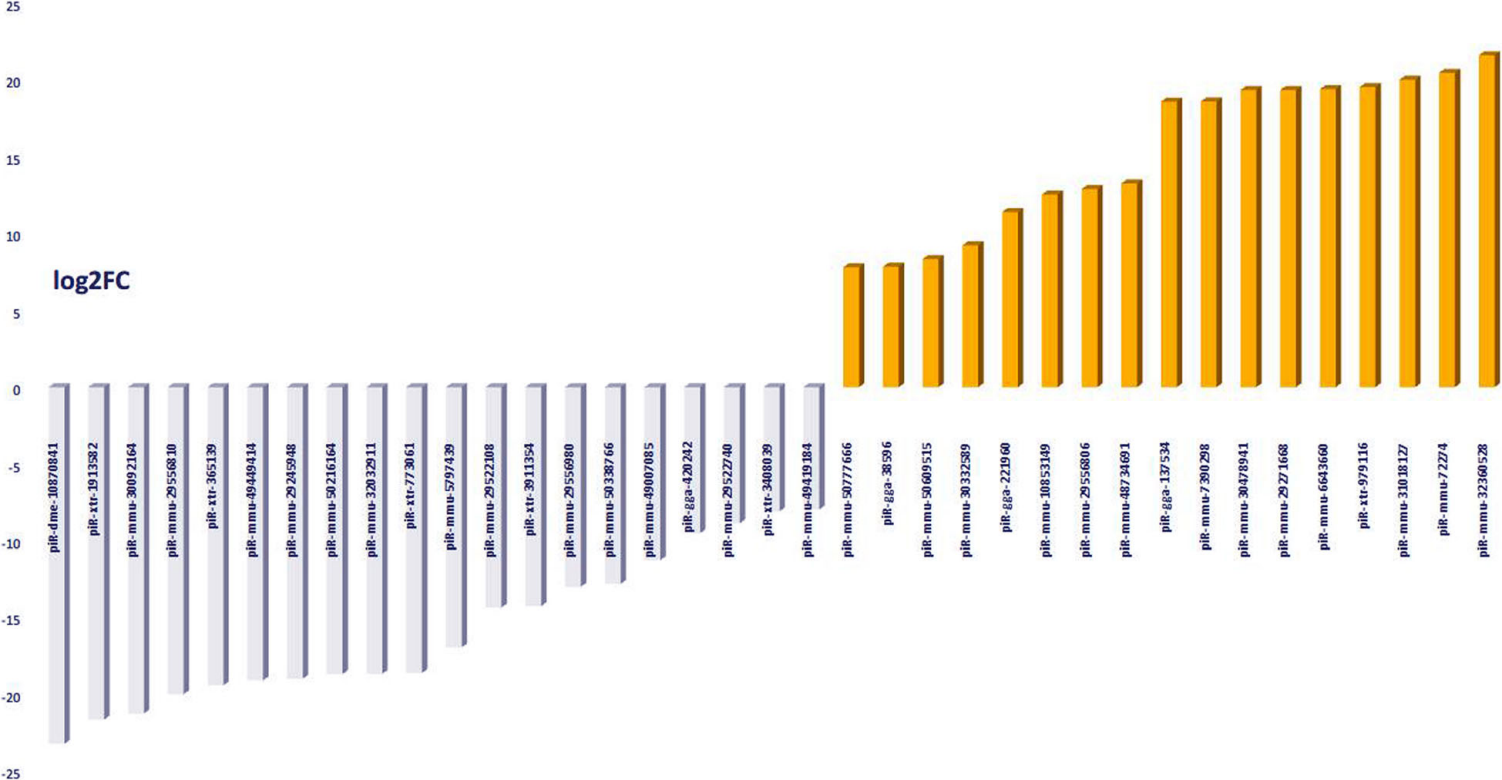
Differential expression of 37 candidate signature piwi interacting RNAs (piRNAs) between male and pseudomale *C. semilaevis* using the TPM (transcript per million) value from small RNA sequencing

**Fig. 5:**
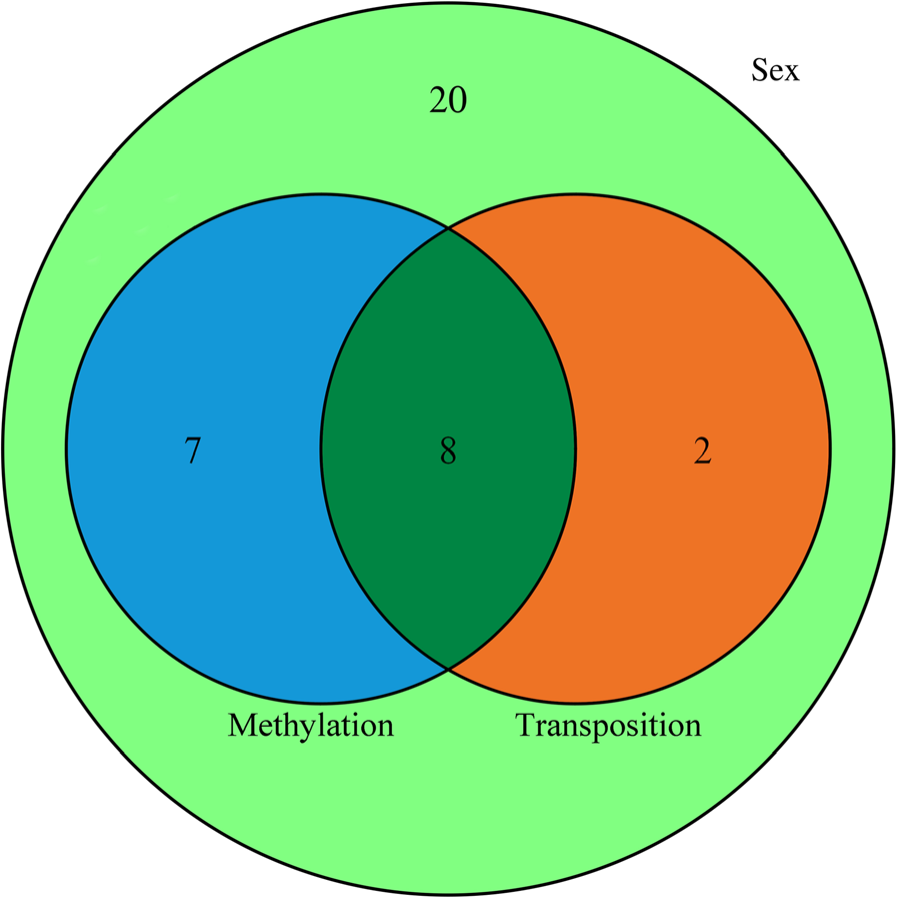
Venn diagram analysis among 37 sex-related, 15 methylation-related, and 10 transposition-related piwi interacting RNAs (piRNAs), showing the intersection of the three sets

The 6231 target genes were employed for gene ontology (GO) enrichment and Kyoto Encyclopedia of Gene and Genomes (KEGG) pathway enrichment analysis. GO term categories generated from the 6231 genes targeted by the 44 candidate signature piRNAs showed that most of the target genes are involved in plasma membrane, integral component of membrane, extracellular exosome, metal ion, and ATP binding, transcription, and DNA − templates in the cellular component and biological process categories (Supplementary Fig. 6). Among the target genes of the 44 candidate signature piRNAs, those related to sex differentiation, sex determination, and sex development in subsystems of GO enrichment and KEGG pathway enrichment analysis were identified, which allowed us to reduce the number of candidate signature piRNAs to 37 (Additional files 6). The 37 candidate signature piRNAs included: 17 piRNAs with high expression in the ZZ♂ group but little expression in ZW♂; and 20 piRNAs with a non-zero expression in the ZZ♂ group, but much higher expression in the ZW♂ group. The expression profiles of these piRNAs are shown in Fig. 4 and Additional file 7. The KEGG pathway enrichment analysis showed that lipid-carbohydrate metabolism and signal transduction were the top two functional categories of the target genes (Supplementary Fig. 7). Meanwhile, we also investigated the target genes related to DNA methylation and transposition, because previous research showed that epigenetic regulation plays multiple crucial roles in the sex reversal of half-smooth tongue sole, and piRNAs may be involved in transposon silencing. Fifty-three DNA methylation related target genes were identified that were predicted to interact with 15 piRNAs (Additional files 8). Meanwhile, 16 target genes related to transposition, especially heterochromatin formation, were also identified together with their 10 interacting piRNAs (Additional files 9). We carried out Venn diagram analysis among the 37 sex-related, 15 methylation-related, and 10 transposition-related piRNAs, which identified eight piRNAs in the intersection of all three sets. The result implied that piRNAs might regulate the sex development of *C. semilaevis* through epigenetic regulation or transposition (Fig. 5).

#### Verification by qRT-PCR

**Fig. 6:**
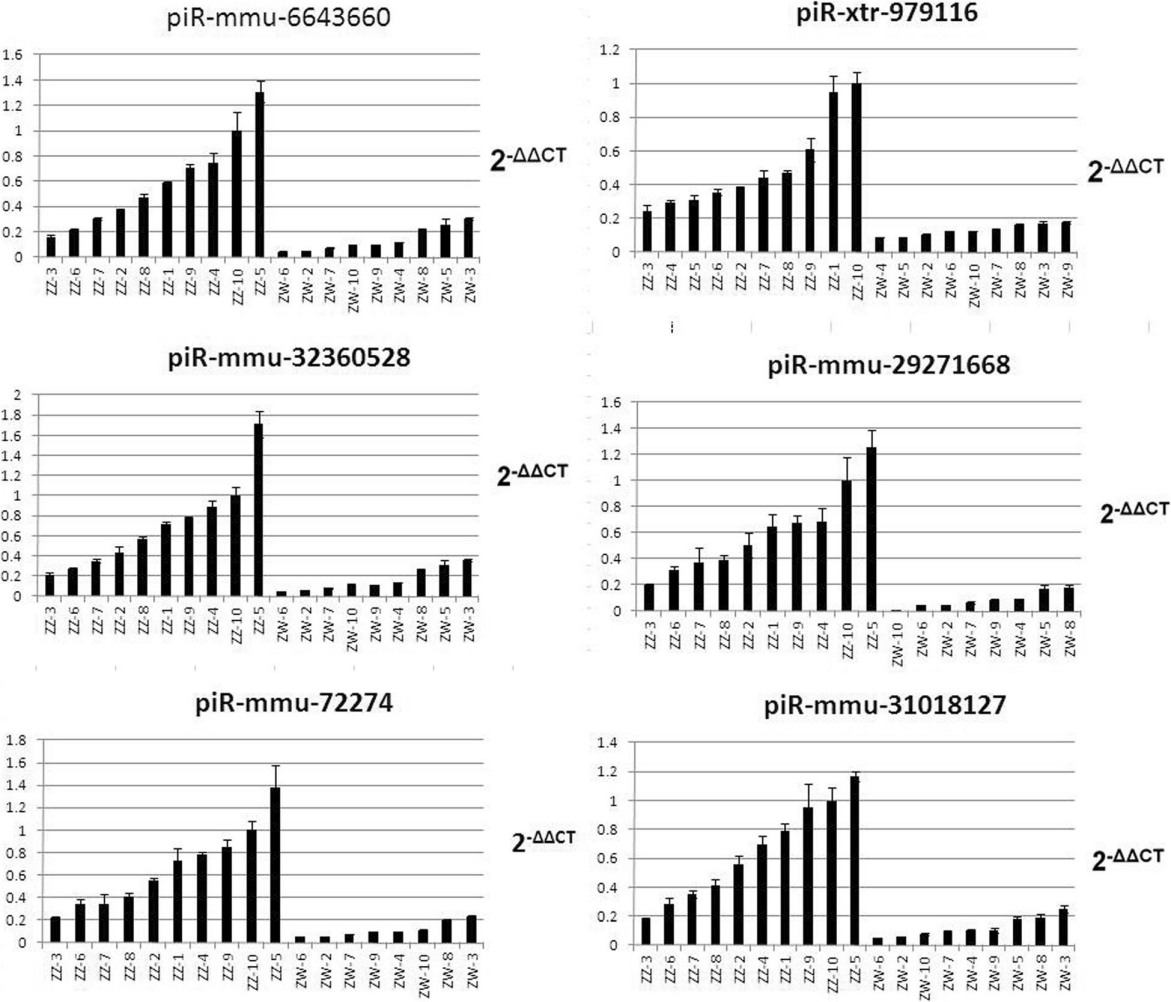
Quantitative real-time reverse transcription PCR (qRT-PCR) to quantitatively verify the expression of marker piwi interacting RNAs (piRNAs) in 10 male (ZZ♂) and 10 pseudomale (ZW♂) *C. semilaevis*

To investigate the candidate signature piRNAs identified in the present study, we chose 15 candidate signature piRNAs for further verification by qRT-PCR from among the 37 most differentially expressed candidate piRNAs. We used RNA from 10 male and 10 pseudomale fish to carry out qRT-PCR to quantitatively measure the expression of marker piRNAs. The results of qRT-PCR showed that the expression of six marker piRNAs in 10 male and 10 pseudomale fish were significantly higher in males than pseudomales (Fig. 6 and Additional file 10), which was consistent with the results obtained from the piRNA profiling in the small RNA sequencing analysis. Therefore, these six signature piRNAs (piR-mmu-29,271,668, piR-mmu-6,643,660, piR-xtr-979,116, piR-mmu-32,360,528, piR-mmu-72,274, and piR-mmu-31,018,127) could be considered as male molecular biomarkers for *C. semilaevis.*

## Discussion

The sex determining mechanisms of fish are complex and diverse. Research using model organisms has revealed that gender determination is influenced by many factors [25]. We chose half-smooth tongue sole as a model to characterize reproductive regulation differences at the subcellular and molecular level between male and pseudomale fish. As a result, several signature biomarkers were developed based on small RNA sequencing. We successfully isolated and captured exosomes derived from seminal plasma in *C. semilaevis*, from which we identified six piRNAs with significant differential expression for development as biomarkers to distinguish males from pseudomales in sex identification.

It is considered important to determine piRNAs’ functions in animal development. PiRNAs are believed to be closely related to reproductive development of mammals. Previous studies have demonstrated that piRNAs are necessary for spermatogenesis in *Caenorhabditis elegans* [26], zebrafish [27], and mouse [28], because piRNA complexes are involved in post-transcriptional gene silencing of transposons. Compared with miRNAs, piRNAs have less conserved sequences and play a more important role in reproductive regulation, especially in testis development and spermatogenesis. Therefore, there are good grounds for developing sex-specific piRNA markers. Previously, several female-specific biomarkers were developed in *C. semilaevis*, including amplified fragment length polymorphism (AFLP markers (CseF382) (accession no. DQ487760) [3] and a co-dominant microsatellite marker (CyseSLM) by screening genomic microsatellites [22], which were developed based on genomic DNA sequences. However, there remains a lack of a suitable male specific molecular marker in the half smooth tongue sole.

Wang et al. used next generation sequencing to develop 289 piRNA clusters (PRCs) generated from the gonad of Japanese flounder (*Paralichthys olivaceus*) as candidate signatures. Finally, seven PRCs were validated as signatures using qRT-PCR [29]. MiRNAs enclosed by exosomes were more commonly employed as biomarkers to diagnosis and identify physiological characteristics. Sun et al. identified seven signature miRNAs derived from serum exosomes between male and female *C. semilaevis* [30]. Our work is the first to use piRNAs from exosomes as biomarkers in fish. We identified six piRNAs with significant differential expression as biomarkers for males in sex identification. Target gene prediction showed that there was a high coincidence between piRNA targets related to sex development and DNA methylation. Earlier research indicated that the piRNA pathway relies on the specificity provided by the piRNA sequence to identify complementary TE targets, while the effector function is provided by the PIWI protein. PIWI silences TE transcription at the chromatin level by directing inhibited histone marker deposition and DNA methylation to the TE copy [31, 32]. Whether the sex regulation mechanism of half smooth tongue sole depends on epigenetic regulation or DNA methylation through transcriptional silencing by piRNAs requires further study.

Our signature piRNAs: piR-mmu-6,643,660, piR-mmu-32,360,528, piR-mmu-72,274, piR-mmu-31,018,127, piR-mmu-29,271,668, and piR-xtr-979,116, were all highly expressed in male *C. semilaevis* donors, but showed very low expression in pseudomale fish. Different piRNAs have performed differently, for example, piR-xtr-979,116 has excellent distinguishability between two groups. However, for some individual, obviously, the differences are not significant. Individuals ZZ 5 and ZZ 10 had the higher expression in all six signatures, while ZZ 3 and ZZ 6 has had lower expression. It should be noted that all the samples were obtained at the same stage, (Random sampling from different ponds in the same factory at same time). We speculated that the environment or individual differences may result in the relatively low expression in ZZ 3 and ZZ 6, and this may only reflect to a certain extent that the piRNAs we selected has certainare representativeness. More individuals are needed to verify the judgment ability of these six markers. Among the six piRNAs, first four signatures are related to both sex development and methylation through target gene prediction, while the last two are related to sex development only. Currently, the functions of the six piRNAs are unclear in all species, including fish, indicating that further studies should be focus on the regulatory mechanism of these piRNA during their interaction with their targets.

## Conclusion

In this study, exosomes derived from *C. semilaevis* seminal plasma were successfully isolated*.* Small RNAs were sequenced and used to identify signature piRNAs as sexual biomarkers to distinguish male and pseudomale *C. semilaevis*. We identified 44 candidate signature piRNAs with extremely significant differential expression profiles. Target genes were predicted and then subjected to GO enrichment and KEGG pathway enrichment analysis. Furthermore, eight piRNAs appeared at the intersection of 37 sex-related, 15 methylation-related, and 10-transposition related piRNAs, which implied that these piRNAs might regulate sex development of *C. semilaevis* through epigenetic regulation or transposition. Finally, six markers that were verified by qRT-PCR were selected as signature miRNAs. This work provides the basis for a method to identify the sex of fish by employing piRNAs derived from exosomes as biomarkers, which might prove to be applicable to other species in the future.

## Materials and methods

### Isolation of exosomes from *C. semilaevis*

*C. semilaevis* specimens were obtained from Weizhuo Ltd., Hebei province, China. As identified by sex molecular markers developed previously by Zhang 2019 [33], 60 male and 60 pseudomale 2-year-old live fish were selected for semen collection. Seminal fluid was collected from 30 male fish and then merged for exosome isolation, as were the samples from 30 pseudomale fish. Before sample collection, we covered the fish’s face with a wet towel to avoid excessive stress. Each fish were collected about 0.2 ml seminal fluid, and then released into seawater pond. No fish need to be anesthetized or euthanized during the whole sample collection. Exosomes were isolated using a Total Exosome Isolation Kit (System Biosciences, Palo Alto, CA, USA) [34, 35]. The isolated exosome pellet was resuspended in 1/10 of the original volume in sterile water for subsequent detection and analysis [36–38].

### Transmission electron microscopy

Exosomes were fixed to formvar-carbon coated 300 mesh copper grids [39]. The absorbed exosomes were then negatively stained with 3% phosphotungstic acid and dried at room temperature for 20 min [40]. Subsequently, the exosomes were observed under a transmission electron microscope (Hitachi, H600IV, Japan) and the images were captured using a digital camera (Sony, Tokyo, Japan) [41–43].

### Nanoparticle tracking analysis

Quantification and size analysis of the purified exosomes (5 μl, three times) were performed using a NanoSight NS300 instrument (Malvern Instruments, Westborough, MA, USA) [44]. Vesicles were visualized using light scattering under a light microscope [45]. Measurement of the exosome concentration was performed by calculating particle size on a particle-by-particle basis in a 60-s video recorded at a frame rate of 25 frames/s to provide accuracy and statistics for further analysis [46, 47]. The results were subsequently analysed using the NTA 2.3 software (Malvern Instruments).

### Western blotting analysis

Exosomal extracts (each sample contained at least 10 μg of total protein) were separated using sodium dodecyl sulphate-polyacrylamide gel electrophoresis (SDS-PAGE) and transferred to polyvinylidene fluoride (PVDF) membranes (Millipore Corp. Bedford, MA, USA). The membranes were blocked in Tris-buffered saline (TBS) containing 5% skimmed milk at 37 °C for 2 h and then incubated with specific primary antibodies (anti-CD63 antibodies, anti-CD9 antibodies, and anti-HSP90 antibodies) at 4 °C overnight (1:1000, System Biosciences, Palo Alto, CA, USA). The membranes were washed three times with TBST (TBS containing 0.1% Tween-20) and then incubated with a peroxidase-labelled anti-rabbit secondary antibody (1:1000, System Biosciences) at 37 °C for 45 min. After washing with TBST three times, the membranes were visualized using chemiluminescence with the enhanced chemiluminescence (ECL) western blot analysis system (Novex ECL Chemiluminescent Substrate, Life Technologies, Carlsbad, CA, USA).

### Small RNA library construction and sequencing

Total RNAs were extracted using the TRIzol reagent (Invitrogen, Waltham, MA, USA). The RNA donors from the two groups were sequenced separately. Small RNA libraries were constructed and sequenced using TruSeq Small RNA Donor Prep Kits (Illumina Inc., San Diego, CA, USA) by the OE Biotech Company (Shanghai, China). The basic reads were converted into sequence data (also called raw data/reads) by base calling. Low-quality reads were filtered out, and the reads with 5′ primer contaminants and poly (A) regions were removed. Reads without a 3′ adapter and insert tag, reads shorter than 15 nt, and those longer than 41 nt were filtered out of the raw data to obtain clean reads.

For primary analysis, the length distribution of the clean sequences in the reference genome was determined. Non-coding RNAs were annotated as ribosomal RNAs (rRNAs), transfer RNAs (tRNAs), small nuclear RNAs (snRNAs), small nucleolar RNAs (snoRNAs), and miRNAs. The known miRNAs were identified by alignment against the miRBase v.21 database (http://www.mirbase.org/) [48, 49], and the unaligned reads were processed for annotation using Rfam (version 10.1) by BLASTn [50]. The remaining unannotated reads were aligned and analysed using piRBase (http://www.regulatoryrna.org/database/piRNA/).

The expression patterns of known piRNAs in the different donors were analysed. Unannotated reads were analysed using Piano [51] to predict novel piRNAs (http://ento.njau.edu.cn/Piano.html). Previous studies showed that by using the structure and sequence characteristics ofnovel piRNAs prediction, Piano demonstrated excellent predictive performance for piRNAs. Differentially expressed piRNAs were identified with the threshold value of *p* < 0.05. The *p* value was calculated using the DEG algorithm [52] in the R package with the Audic-Claverie statistic [53] without biological replicates. The targets of the differentially expressed piRNAs were predicted using the MiRanda software [54] for animals, with the following parameters: S ≥ 150; ΔG ≤ − 30 kcal/mol and strict 5′ seed pairing. Differentially expressed piRNAs were screened out according to the criteria detailed in the methods section shown in Additional file 4).GO enrichment and KEGG pathway enrichment analysis of differentially expressed piRNA-target genes were performed using R based on the hypergeometric distribution.

### Real-time quantitative PCR to verify signature piRNAs expression

Exosomal piRNA expression was assayed using real qRT-PCR. Total RNAs (20 ng) from the two donor groups were used for reverse transcription. The RT reactions from total RNA were performed using specific Taqman MicroRNA primers (shown in the Additional file 11) and a thermal cycler under the following conditions: 30 min at 65 °C, 50 min at 42 °C, and 5 min at 95 °C. The products were stored at − 20 °C for later use or immediately processed according to the manufacturer’s protocol. Quantitative PCR was performed in 96-well reaction plates using a QuantStudio6 Flex Real-Time PCR System (Thermo Fisher Scientific). No template controls were used to evaluate background signal. The qPCR program consisted of 95 °C for 5 min, followed by 40 cycles each of denaturation at 95 °C for 5 s and annealing and extension for 30 s at 60 °C. The expression level of U6 was used as a stable endogenous control for normalization. Each donor sample was run in triplicate and the relative quantification of piRNA expression was calculated using the 2^−ΔΔCt^ method [55].

## Supplementary information

**Additional file 1 **List of 14,006 non repetitive novel piRNAs predicted from both pseudomales (ZW) and male (ZZ) donors of *Cynoglossus semilaevis*.

**Additional file 2.** List of 7070 novel piRNAs categories in ZZ donorgroup.

**Additional file 3.** List of 11,588 novel piRNAs categories in ZW donor group

**Additional file 4.** List of 26,135 differentially expressed piRNAs identified according to the criteria detailed in the methods section in the males and pseudomales.

**Additional file 5.** List of Target genes of the 44 candidate signature piRNAs that differentially expressed in males and pseudo males.

**Additional file 6.** List of 37 candidate signature piRNAs captured from 44 candidate signature after sex related gene ontology (GO) enrichment and Kyoto Encyclopedia of Gene and Genomes (KEGG) pathway enrichment analysis.

**Additional file 7.** The expression profiles of 37 signature piRNAs by small RNA sequencing in males and pseudomales

**Additional file 8.** List of 53 DNA methylation related target genes identified that predicted to interact with 15 regulating piRNAs

**Additional file 9.** List of 16 target genes related to transposition, especially heterochromatin formation identified together with their 10 interacting piRNAs.

**Additional file 10.** The RT-qPCR datas of the expressions of all six piRNA markers

**Additional file 11.** List of piR primers of six piRNAs in the RT-qPCR

**Additional file 12 Supplementary Figure 1**. CD63 blot. 1.ZZ;2. ZW; 3. other exosome sample1; 4.other exosome sample2; 5. serum exosome sample; 6. Negative control (cell sample). **Supplementary Figure 2**. HSP90 blot;1.ZZ; 2. ZW; 3. other exosome sample1; 4.other exosome sample2; 5. serum exosome sample; 6. Negative control (cell sample). **Supplementary Figure 3**. CD9 blot. **Supplementary Figure 4**. Statistics of length distribution of small RNA from next generation Sequencing (NGS) In two donor groups: the peak values for both groups were mainly concentrated on 31 bp.ZW, male; ZZ, pseudomale. Blue line represents sample ZZ; Orange line represents sample ZW; The peak value of the curve both appeared at 31 bp. **Supplementary Figure 5**. Screening process for the differential expression of piwi interacting RNAs (piRNAs) between two donor groups. (piRNA, piwi interacting RNA, TPM, transcript per million; GO, gene ontology; KEG, Kyoto Encyclopedia of Genes and Genomes). **Supplementary Figure 6**. Gene ontology (GO) enrichment analysis of 37 candidate signature piwi interacting RNAs (piRNAs) target genes. The figure shows that top30 target genes of 37 piRNAs were represented as results of BP, CC and MF respectively TOP10. **Supplementary Figure 7**. Kyoto Encyclopedia of Genes and Genomes (KEGG) enrichment analysis of 37 candidate signature piwi interacting RNA (piRNA) target genes.

## Data Availability

All sequence alignment and screened out data are presented in format in Additional files 1,2, 3, 4, 5, 6, 7, 8, 9, 10, 11.

## Abbreviations

*C. semilaevis*: *Cynoglossus semilaevis*
piRNAs: Piwi-interacting RNAs
miRNAs: MicroRNAs
TE: Transposon element
AFLP: Amplified fragment-length polymorphism
SNPs: Single nucleotide polymorphisms
qRT-PCR: Quantitative real-time reverse transcription PCR
TEM: Transmission electron microscopy
NTA: Nanoparticle tracking analysis
TPM: Transcript per million
GO: Gene ontology
KEGG: Kyoto Encyclopedia of Gene and Genomes
CyseSLM: Co-dominant microsatellite marker
PRCs: piRNA clusters
SDS-PAGE: Sodium dodecyl sulphate-polyacrylamide gel electrophoresis
PVDF: Polyvinylidene fluoride
TBS: Tris-buffered saline
ECL: Enhanced chemiluminescence
rRNAs: Ribosomal RNAs
tRNAs: Transfer RNAs
snRNAs: Small nuclear RNAs
