# Supplementary material for: Identification and application of piwi-interacting RNAs from seminal plasma exosomes in Cynoglossus semilaevis
Source: BMC Genomics. 2020 Apr 15;21:302. doi: 10.1186/s12864-020-6660-7 (PMC7158113; doi:10.1186/s12864-020-6660-7)
Supplement: Supplementary file 11 — Additional file 11. List of piR primers of six piRNAs in the RT-qPCR [file 12864_2020_6660_MOESM11_ESM.doc]

| **Gene**  U6  piR-mmu-6643660 | **Primers** | **Sequence** | **Product size**  93 bp |
| --- | --- | --- | --- |
|  | U6-F  U6-R  piR-mmu-6643660-F  piR-mmu-6643660-R | CTCGCTTCGGCAGCACATATACT  ACGCTTCACGAATTTGCGTGTC  CATTGGTGGTTCAGTGGTAGAA  TCGTATCCAGTGCAGGGTC |  |
|  | piR-mmu-6643660-RT | GTCGTATCCAGTGCAGGGTCCGAGGTATTCGCACTGGATACGAGGCGAGAA | 72bp |
| piR-xtr-979116 | piR-xtr-979116-F | TTAATCGGGTTCGTTTCCC | 73bp |
|  | piR-xtr-979116-R | TCGTATCCAGTGCAGGGTC |  |
|  | piR-xtr-979116-RT | GTCGTATCCAGTGCAGGGTCCGAGGTATTCGCACTGGATACGATGGTGCGT |  |
| piR-mmu-32360528 | piR-mmu-32360528-F | AGCATTG GTGGTTCAGTGG | 72bp |
|  | piR-mmu-32360528-R | TCGTATCCAGTGCAGGGTC |  |
|  | piR-mmu-32360528-RT | GTCGTATCCAGTGCAGGGTCCGAGGTATTCGCACTGGATACGAAGAGAATT |  |
| piR-mmu-29271668 | piR-mmu-29271668-F | GCATTGGTGGTTCAGTGGT | 72bp |
|  | piR-mmu-29271668-R | TCGTATCCAGTGCAGGGTC |  |
|  | piR-mmu-29271668- RT | GTCGTATCCAGTGCAGGGTCCGAGGTATTCGCACTGGATACGATCCAAAAT |  |
| piR-mmu-72274 | piR-mmu-72274-F | GCATTGGTGGTTCAGTGGT | 74bp |
|  | piR-mmu-72274-R | TCGTATCCAGTGCAGGGTC |  |
|  | piR-mmu-72274- RT | GTCGTATCCAGTGCAGGGTCCGAGGTATTCGCACTGGATACGAAGGCGAGA |  |
| piR-mmu-31018127 | piR-mmu-31018127-F | TACGCATGTGGTTCAGTGGT | 70bp |
|  | piR-mmu-31018127-R | TCGTATCCAGTGCAGGGTC |  |
|  | piR-mmu-31018127- RT | GTCGTATCCAGTGCAGGGTCCGAGGTATTCGCACTGGATACGACGGCGAGA |  |
