# Supplementary material for: Identification and application of piwi-interacting RNAs from seminal plasma exosomes in Cynoglossus semilaevis
Source: BMC Genomics. 2020 Apr 15;21:302. doi: 10.1186/s12864-020-6660-7 (PMC7158113; doi:10.1186/s12864-020-6660-7)

Supplementary Figures

Identification and application of piwi-interacting RNAs from seminal plasma exosome in *Cynoglossus semilaevis*

Bo Zhang[# 1,2], Na zhao[# 3], Lei Jia[#2], Jinyuan Che[1] , Xiaoxu He[2], Kefeng Liu[2], Baolong Bao[* 1]

[1] Key Laboratory of Exploration and Utilization of Aquatic Genetic Resources (Shanghai Ocean University), Ministry of Education; International Research Center for Marine Biosciences at Shanghai Ocean University, Ministry of Science and Technology; National Demonstration Center for Experimental Fisheries Science Education, Shanghai Ocean University, Shanghai 201306, China. [2]Tianjin Bohai Sea Fisheries Research Institute, Tianjin, China. [3] Tianjin Haolinsaiao Biotechnology Co, Ltd, Tianjin, China.

[#] These authors contributed equally to this work.

* Corresponding author.

Figure legend

We separated the membrane to three parts following the guide of prestained protein marker after blocking. The three parts were then incubated with different antibodies respectively.

Supplementary Fig 1: CD63 blot. 1.ZZ;2. ZW; 3. other exosome sample1; 4.other exosome sample2; 5. serum exosome sample; 6. Negative control（ cell sample）

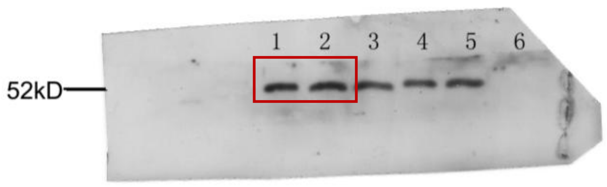

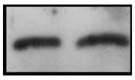

Supplementary Fig 2: HSP90 blot;1.ZZ; 2. ZW; 3. other exosome sample1; 4.other exosome sample2; 5. serum exosome sample; 6. Negative control（ cell sample）

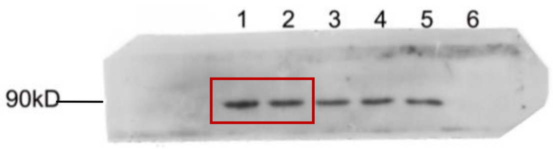

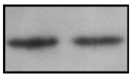

Supplementary Fig 3: CD9 blot

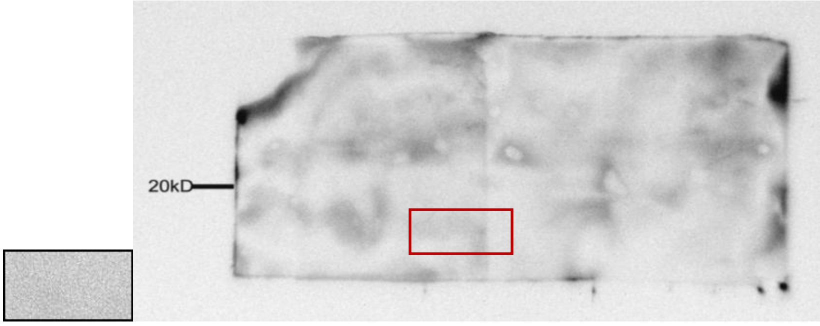

Supplementary Fig4:

Statistics of length distribution of small RNA from next generation sequencing (NGS) in two donor groups:

the peak values for both groups were mainly concentrated on 31 bp.ZW, male; ZZ, pseudo-male. Blue line represents sample ZZ; Orange line represents sample ZW; The peak value of the curve both appeared at 31bp

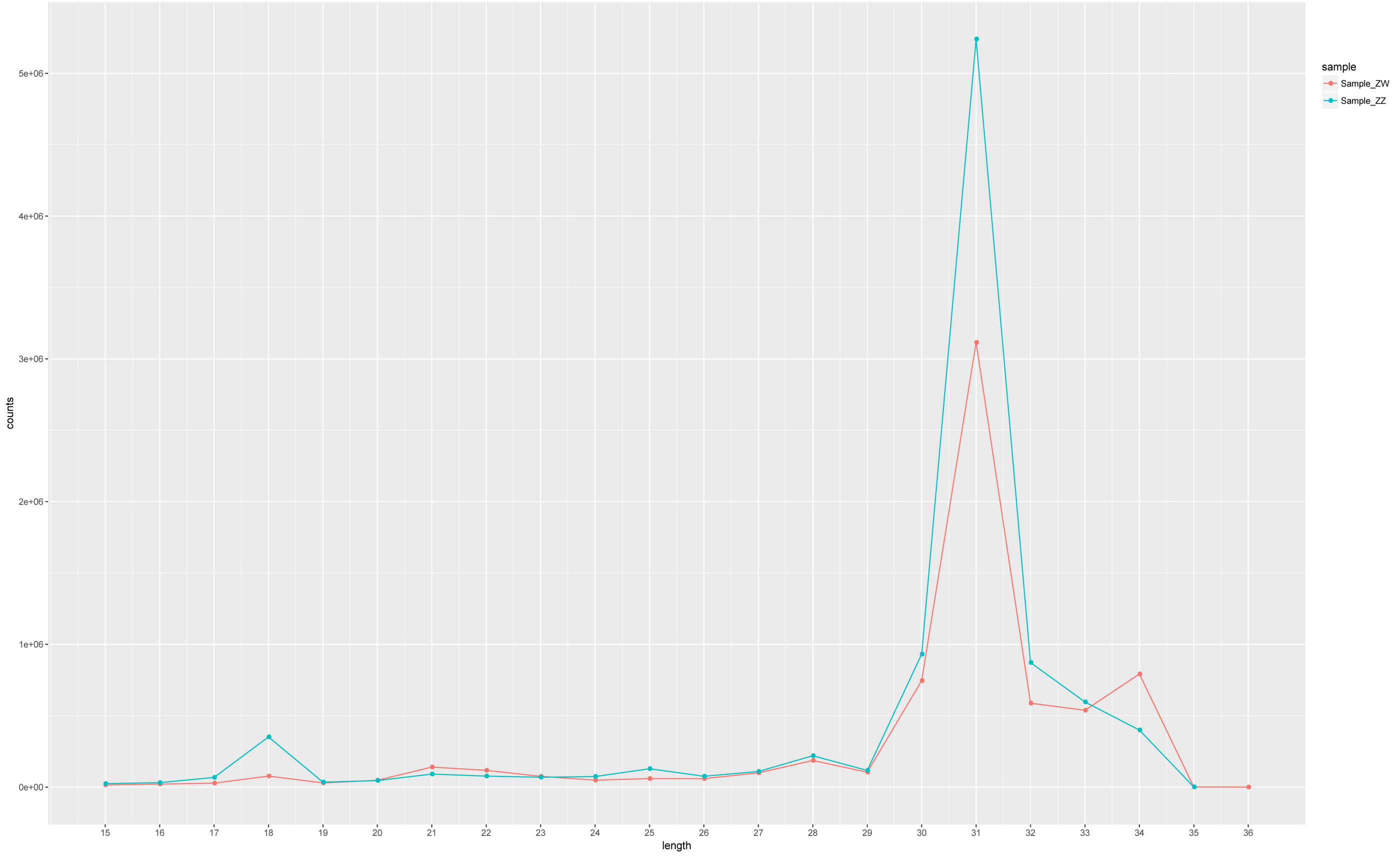

Supplementary Fig 5: Screening process for the differential expression of piwi interacting RNAs (piRNAs) between two donor groups. (piRNA, piwi interacting RNA, TPM, transcript per million; GO, gene ontology; KEG, Kyoto Encyclopedia of Genes and Genomes)

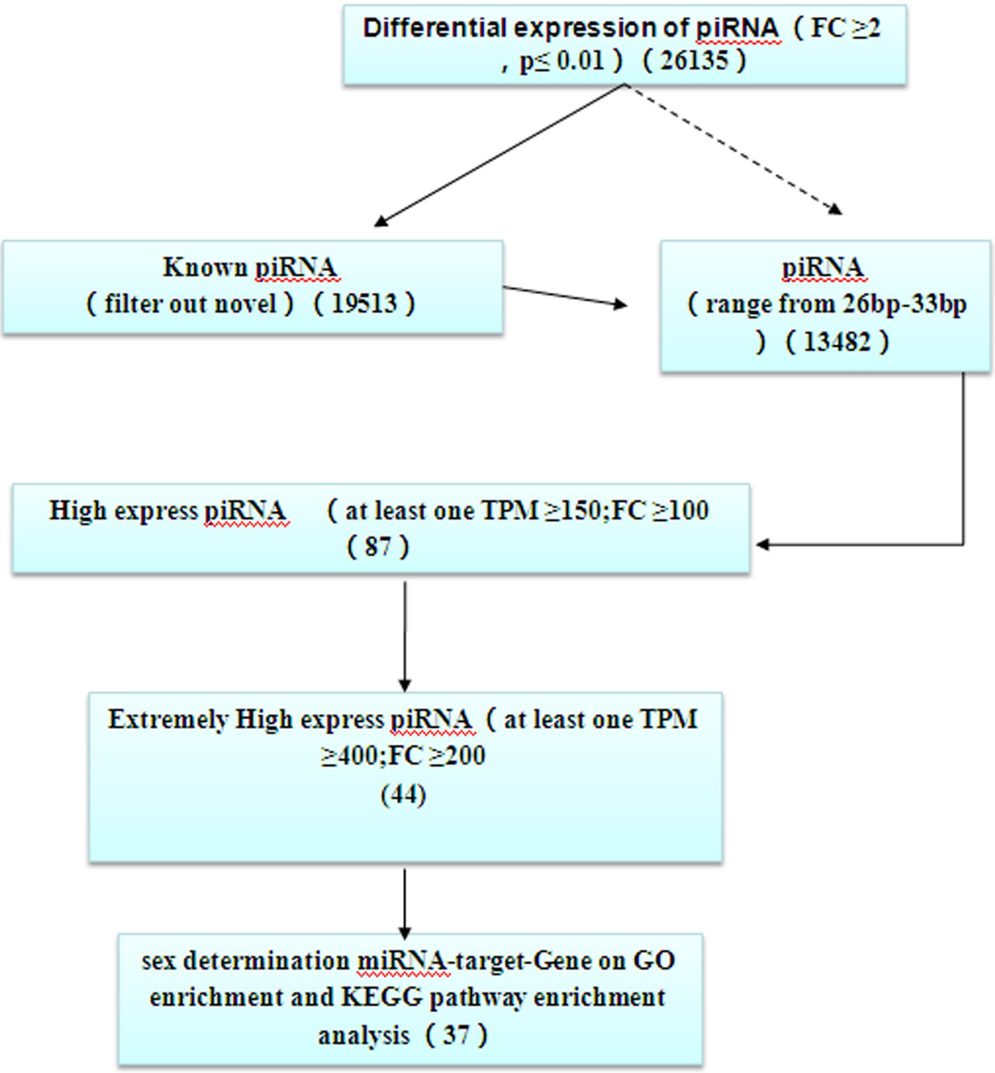

Supplementary Fig 6.

Gene ontology (GO) enrichment analysis of 37 candidate signature piwi interacting RNAs (piRNAs) target genes. The figure shows that top30 target genes of 37 piRNAs were represented as results of BP, CC and MF respectively TOP10 .

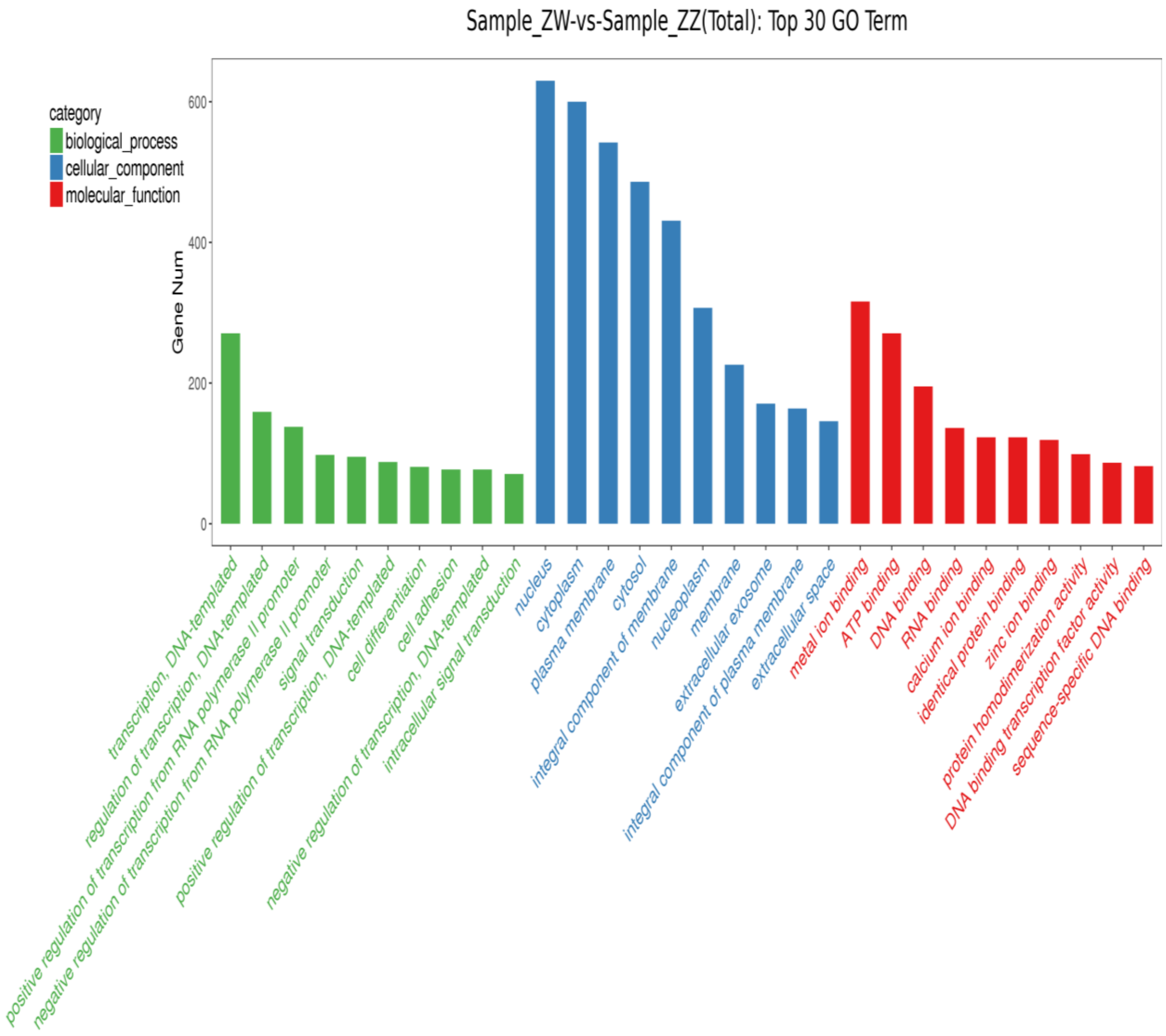

Supplementary Fig 7. Kyoto Encyclopedia of Genes and Genomes (KEGG) enrichment analysis of 37 candidate signature piwi interacting RNA (piRNA) target genes.

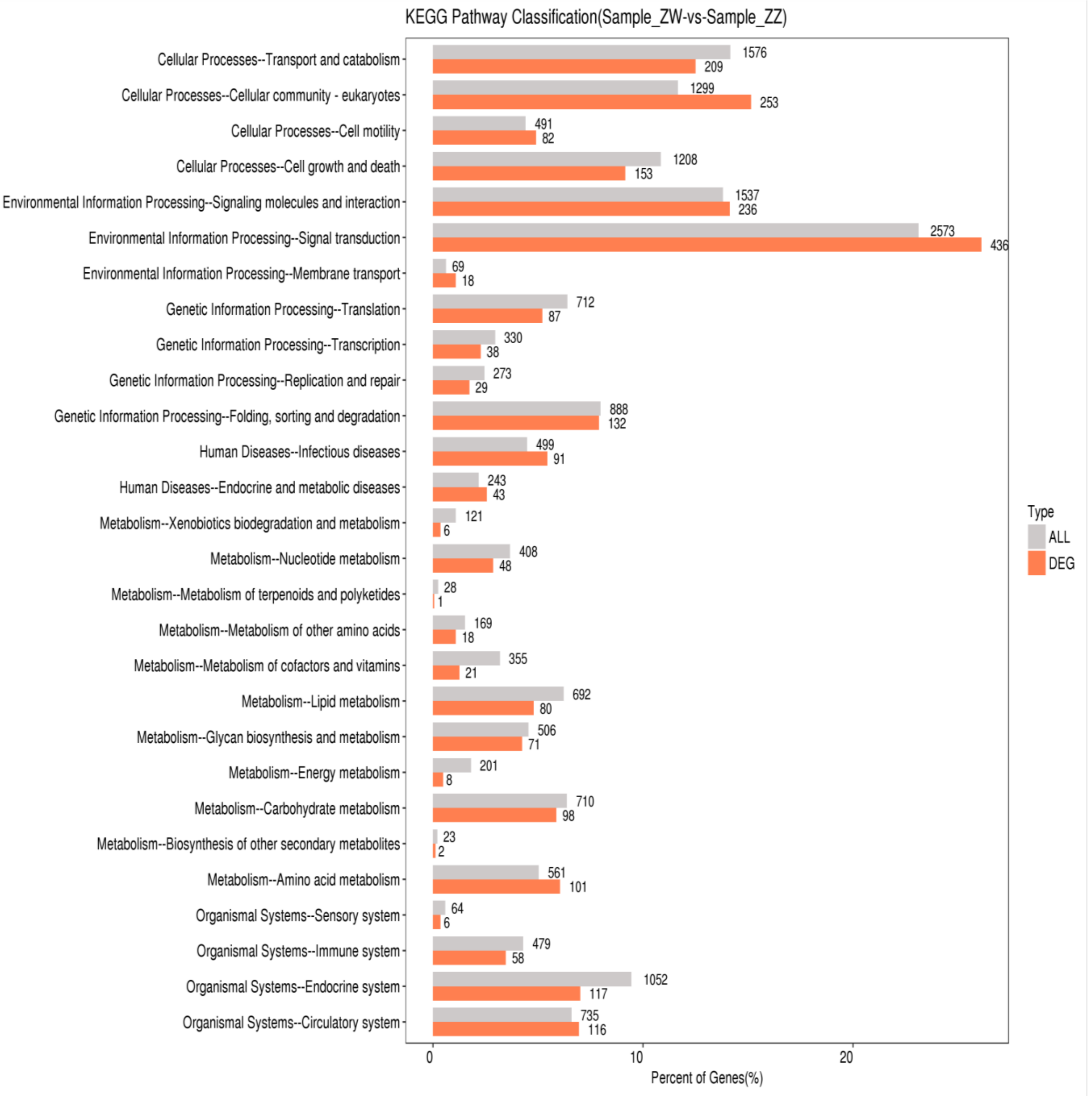

Supplement: Supplementary file 12 — Additional file 12 Supplementary Figure 1. CD63 blot. 1.ZZ;2. ZW; 3. other exosome sample1; 4.other exosome sample2; 5. serum exosome sample; 6. Negative control (cell sample). Supplementary Figure 2. HSP90 blot;1.ZZ; 2. ZW; 3. other exosome sample1; 4.other exosome sample2; 5. serum exosome sample; 6. Negative control (cell sample). Supplementary Figure 3. CD9 blot. Supplementary Figure 4. Statistics of length distribution of small RNA from next generation Sequencing (NGS) In two donor groups: the peak values for both groups were mainly concentrated on 31 bp.ZW, male; ZZ, pseudomale. Blue line represents sample ZZ; Orange line represents sample ZW; The peak value of the curve both appeared at 31 bp. Supplementary Figure 5. Screening process for the differential expression of piwi interacting RNAs (piRNAs) between two donor groups. (piRNA, piwi interacting RNA, TPM, transcript per million; GO, gene ontology; KEG, Kyoto Encyclopedia of Genes and Genomes). Supplementary Figure 6. Gene ontology (GO) enrichment analysis of 37 candidate signature piwi interacting RNAs (piRNAs) target genes. The figure shows that top30 target genes of 37 piRNAs were represented as results of BP, CC and MF respectively TOP10. Supplementary Figure 7. Kyoto Encyclopedia of Genes and Genomes (KEGG) enrichment analysis of 37 candidate signature piwi interacting RNA (piRNA) target genes. [file 12864_2020_6660_MOESM12_ESM.pdf]
